# Supplementary material for: De novo fatty-acid synthesis protects invariant NKT cells from cell death, thereby promoting their homeostasis and pathogenic roles in airway hyperresponsiveness
Source: eLife. 2023 Nov 2;12:RP87536. doi: 10.7554/eLife.87536 (PMC10622147; doi:10.7554/eLife.87536)
Supplement: Supplementary file 2. [file elife-87536-supp2.docx]

| Species | Gene | F/R | Sequence |
| --- | --- | --- | --- |
| Mouse | *Acox1* | F | CTG CCA AGG GAC TCC AGA GCA GCT |
| Mouse | *Acox1* | R | GAC ATG GAC ACA TCC ACC ATG CAG |
| Mouse | *Fasn* | F | AGC GGC CAT TTC CAT TGC CC |
| Mouse | *Fasn* | R | CCA TGC CCA GAG GGT GGT TG |
| Mouse | *Acc1* | F | ACA GTG GAG CTA GAA TTG GAC |
| Mouse | *Acc1* | R | ACT TCC CGA CCA AGG ACT TTG |
| Mouse | *Pparg* | F | GTG ATG GAA GAC CAC TCG CAT T |
| Mouse | *Pparg* | R | CCA TGA GGG AGT TAG AAG GTT C |
| Mouse | *Tbx21* | F | TTCCCATTCCTGTCCTTCAC |
| Mouse | *Tbx21* | R | CCACATCCACAAACATCCTG |
| Mouse | *Gata3* | F | GGAAACTCCGTCAGGGCTA |
| Mouse | *Gata3* | R | AGAGATCCGTGCAGCAGAG |
| Mouse | *Rorc* | F | TGA GGC CAT TCA GTA TGT GG |
| Mouse | *Rorc* | R | CTT CCA TTG CTC CTG CTT TC |
| Mouse | *Foxp3* | F | CCC AGG AAA GAC AGC AAC CTT |
| Mouse | *Foxp3* | R | TTC TCA CAA CCA GGC CAC TTG |
| Mouse | *Hk2* | F | AGA GAA CAA GGG CGA GGA G |
| Mouse | *Hk2* | R | GGA AGC GGA CAT CAC AAT C |
| Mouse | *G6pc1* | F | CCA TGC AAA GGA CTA GGA ACA A |
| Mouse | *G6pc1* | R | TAC CAG GGC CGA TGT CAA C |
| Mouse | *Fbp1* | F | CCA TCA TAA TCG AAC CTG AG |
| Mouse | *Fbp1* | R | CTT CTC AGA AGG CTC ATC AG |
| Mouse | *Sdhb* | F | CTA AAT AAG TGC GGA CCT ATG G |
| Mouse | *Sdhb* | R | AGT ATT GCC TCC GTT GAT GTT C |
| Mouse | *Cpt1a* | F | CCA TCC TGT CCT GAC AAG GTT TAG |
| Mouse | *Cpt1a* | R | CCT CAC TTC TGT TAC AGC TAG CAC |
| Mouse | *Pkm2* | F | CTG GCT CAG AAG ATG ATG ATC G |
| Mouse | *Pkm2* | R | CTT GGT GAG CAC GAT AAT GG |
| Mouse | *Cebpa* | F | CAA AGC CAA GAA GTC GGT GGA |
| Mouse | *Cebpa* | R | TCA TTG TGA CTG GTC AAC TCC AGC |
| Mouse | *Bak1* | F | ATA TTA ACC GGC GCT ACG AC |
| Mouse | *Bak1* | R | AGG CGA TCT TGG TGA AGA GT |
| Mouse | *Bax* | F | TAG CAA ACT GGT GCT CAA |
| Mouse | *Bax* | R | TCT TGG ATC CAG ACA AGC AG |
| Mouse | *Bcl2* | F | CTC GTC GCT ACC GTC GTG ACT TCG |
| Mouse | *Bcl2* | R | CAG ATG CCG GTT CAG GTA CTC AGT C |
| Mouse | *Bclxl* | F | TGG AGT AAA CTG GGG GTC GCA TCG |
| Mouse | *Bclxl* | R | AGC CAC CGT CAT GCC CGT CAG G |
| Mouse | *Actb* | F | GGGAAGCTCACTGGCATGG |
| Mouse | *Actb* | R | CTTCTTGATGTCATCATACTTGGCAG |
| Mouse | *Ifng* | F | CGG CAC AGT CAT TGA AAG CCT A |
| Mouse | *Ifng* | R | GTT GCT GAT GGC CTG ATT GTC |
| Mouse | *Il4* | F | TCA ACC CCC AGC TAG TTG TC |
| Mouse | *Il4* | R | TGT TCT TCG TTG CTG TGA GG |
| Mouse | *Il5* | F | CTC TGT TGA CAA GCA ATG AGA CG |
| Mouse | *Il5* | R | TCT TCA GTA TGT CTA GCC CCT G |
| Mouse | *Il13* | F | CCT GGC TCT TGC TTG CCT T |
| Mouse | *Il13* | R | GGT CTT GTG TGA TGT TGC TCA |
| Mouse | *Il17a* | F | GGT CTT GTG TGA TGT TGC TCA |
| Mouse | *Il17a* | R | GGG TCT TCA TTG CGG TGG AGA G |
| Mouse | *Fabp1* | F | AGG AGT GCG AAC TGG AGA CCA T |
| Mouse | *Fabp1* | R | GTC TCC ATT GAG TTC AGT CAC GG |
| Mouse | *Fabp3* | F | AGA GTT CGA CGA GGT GAC AGC A |
| Mouse | *Fabp3* | R | TTG TCT CCT GCC CGT TCC ACT T |
| Mouse | *Fabp5* | F | GAC GAC TGT GTT CTC TTG TAA CC |
| Mouse | *Fabp5* | R | TGT TAT CGT GCT CTC CTT CCC G |
| Human | *PPARG* | F | AGC CTG CGA AAG CCT TTT GGT G |
| Human | *PPARG* | R | GGC TTC ACA TTC AGC AAA CCT GG |
| Human | *ACTB* | F | TCC CTG GAG AAG AGC TAC GA |
| Human | *ACTB* | R | AGC ACT GTG TTG GCG TAC AG |
| Human | *ACC1* | F | ATG GGC GGA ATG GTC TCT TTC |
| Human | *ACC1* | R | TGG GGA CCT TGT CTT CAT CAT |
| Human | *FASN* | F | GGA GGT GGT GAT AGC CGG TAT |
| Human | *FASN* | R | GGG TAA TCC ATA GAG CCC AG |
| Human | *HK2* | F | GAG TTT GAC CTG GAT GTG GTT GC |
| Human | *HK2* | R | CCT CCA TGT AGC AGG CAT TGC T |
| Human | *IL4* | F | CCG TAA CAG ACA TCT TTG CTG CC |
| Human | *IL4* | R | GAG TGT CCT TCT CAT GGT GGC T |
| Human | *IL13* | F | ACG GTC ATT GCT CTC ACT TGC C |
| Human | *IL13* | R | CTG TCA GGT TGA TGC TCC ATA CC |
| Human | *IFNG* | F | GAG TGT GGA GAC CAT CAA GGA AG |
| Human | *IFNG* | R | TGC TTT GCG TTG GAC ATT CAA GTC |
| Human | *IL10* | F | TCT CCG AGA TGC CTT CAG CAG A |
| Human | *IL10* | R | TCA GAC AAG GCT TGG CAA CCC A |
